# A new genus *Vittaliana* belonging to the tribe Opsiini (Hemiptera: Cicadellidae) from India and its molecular phylogeny

**DOI:** 10.7717/peerj.9515

**Published:** 2020-08-27

**Authors:** Sunil, Naresh M. Meshram, Tahseen Raza Hashmi, Pathour R. Shashank

**Affiliations:** National Pusa Collection, Division of Entomology, Indian Council of Agricultural Research-Indian Agricultural Research Institute, New Delhi, Delhi, India

**Keywords:** Leafhoppers, Morphology, Vittaliana, Reticulata, Opsiina, Opsiini, Phylogeny, Deltocephalinae, Hemiptera

## Abstract

The new leafhopper genus *Vittaliana****reticulata* gen. nov., sp. nov., is described from India, and placed in the tribe Opsiini based on ocelli close to eyes, without carina on anterior margin of the face and bifurcate aedeagus with two gonopores. Phylogenetic analysis with maximum likelihood (ML) using IQtree v1.4.1 of combined data (Histone *H3* and *28S* rDNA) reveals that the new genus *Vittaliana* belongs to a clade consisting of *Opsius versicolor* (Distant, 1908), *Opsiini* gen. sp., *Libengaia* sp., *Hishimonus phycitis* (Distant, 1908) and *Yinfomibus menglaensis* Du, Liang & Dai (2019) with good branch support, and that the tribe Opsiini is paraphyletic. This resolves the placement of a new genus in the tribe Opsiini under Deltocephalinae.

## Introduction

The Cicadellide is the largest family of the suborder Auchenorrhyncha, and the Deltocephalinae is the largest and most economically important subfamily of leafhoppers, including at least 6700 described species grouped into 39 tribes (Zahniser & Dietrich, 2013; Dai et al., 2017). The tribe Opsiini is distinguished from other tribes by the face oblique, not strongly depressed, not concave in profile; anterior margin of the head without carinae; antennal bases near middle or posteroventral (lower) corner of eyes; gena not extended onto dorsum behind eyes; the stem of connective longer and bifurcated aedeagus with two gonopores (Emeljanov, 1962; Zahniser & Dietrich, 2013). This tribe is economically important as vectors of viral, bacterial, phytoplasma, and spiroplasma phytopathogens (Nielson, 2002). Zahniser & Dietrich (2013) revised the classification of Deltocephalinae based on molecular and morphological data, and provided a revised interpretation of Opsiini with four subtribes Achaeticina, Circuliferina, Eremophlepsiina, and Opsiina. These subtribes comprises of 40 genera, out of which 29 genera belong to Opsiina with more than 230 species worldwide. The subtribe Opsiina can be differentiated from the others by ovipositor not protruding far beyond pygofer apex and subgenital plates with a lateral row of macrosetae; aedeagal shafts divided near to base (Zahniser & Dietrich, 2013).

Work on this group since Viraktamath, Murthy & Viraktamath (1987); Mitjaev (2000); Dai, Viraktamath & Zhang (2010a); Dai, Dietrich & Zhang (2011); Stiller (2012); El-Sonbati, Wilson & Dhafer (2016), El-Sonbati, Wilson & Al Dhafer (2017), El-Sonbati, Wilson & Dhafer (2020); Fletcher & Dai (2018); Du, Liang & Dai (2019) has led to description of many new taxa. The Old World fauna is known only from regional works (Mitjaev, 2000; Viraktamath & Murthy, 2014; Meshram & Chaubey, 2016). Opsiina contains 29 known genera worldwide, including 10 genera from India so far. In the present work, we describe the *Vittaliana reticulata* **gen. nov., sp. nov.,** and we discuss its phylogenetic position within Deltocephalinae, based on analysis of histone (*H3)* and large ribosomal unit (*28S)* sequences.

## Material and Methods

### For morphological studies

Data was collected as previously described in Meshram, Shashank & Sinha (2017) specifically, in and around ICAR research institutes, Vittal, Kasargod (Kerala: India), with a mercury vapor lamp. Hence, no specific permissions were required for any of the collection localities/activities. Specimens were processed by a series of steps like sorting, cleaning, and mounting. Male genitalia dissections were carried out as described by Oman (1949) and Knight (1965) as follows, the abdomen was removed by inserting a sharp pin between the abdomen and thorax with gentle piercing. The abdomen was treated in 10% KOH for 2∼4 h to remove unsclerotized material by gently prodding the abdomen with the head of a pin. Afterward, the abdomen was rinsed thoroughly in water. The internal structures were then removed by a hooked pin, before being stored in glycerol vials for study.

Photographs were taken with a Leica DFC 425C digital camera on the Leica M205FA stereo zoom automontage microscope.

Repository of the Material: The holotype and paratypes are deposited in National Pusa Collection (NPC), Indian Council of Agricultural Research-Indian Agricultural Research Institute (IARI)-New Delhi, India (with repository number: Holotype: RRS2; Paratypes: RRS3, RRS4, RRS5)

**New Taxon LSID.** *Vittaliana*: urn:lsid:zoobank.org:act:51DA3683-0359-444F-8C11-F630518D8506, *Vittaliana reticulata*: urn:lsid:zoobank.org:act:E690A7AF-7FCD-4460-93BC-1274D932C5F4.

The electronic version of this article in Portable Document Format (PDF) will represent a published work according to the International Commission on Zoological Nomenclature (ICZN), and hence the new names contained in the electronic version are effectively published under that Code from the electronic edition alone. This published work and the nomenclatural acts it contains have been registered in ZooBank, the online registration system for the ICZN. The ZooBank LSIDs (Life Science Identifiers) can be resolved and the associated information viewed through any standard web browser by appending the LSID to the prefix http://zoobank.org/. The LSID for this publication is: urn:lsid:zoobank.org:pub:228D17FC-590C-41C7-9434-60554F753DBA. The online version of this work is archived and available from the following digital repositories: PeerJ, PubMed Central, and CLOCKSS.

### Molecular studies

#### DNA extraction and PCR amplification

The DNA was extracted from the head and thorax of specimens according to manufacturer protocols using DNASure^^®^^ Tissue Mini Kit. The isolated DNA was stored at −20 °C until required. The amplification of the desired product was done with the help diagnostic PCR reactions, using universal histone *H3* primers: HEXAF (forward) 5′-ATGGCTCGTACCAAGCAGACGGC-3′ and HEX- AR (reverse) 5′-ATATCCTTGGGCATGATGGTGAC-3′ (Ogden & Whiting, 2003) and *28S* rDNA primers (for D2 region 5′-AGTCGKGTTGCTTGAKAGTGCAG-3′& 5′-TTCGGGTCCCAACGTGTACG-3′) and for D9–D10 region 5′-GTAGCCAAATGCCTCGTCA-3′&5′-CACAATGATAGGAAGAGCC-3′ (Dietrich et al., 2001). The PCR protocol for Histone *H3* was followed from Zhaniser & Dietrich (2010) under the following cycling protocol: 4 min at 94 °C, 35 cycles of denaturation for 30 s at 94 °C, annealing for 60 s at 47 °C, elongation for 50 s at 72 °C and a final extension 72 °C for 8 min in a C1000™ Thermal cycler.

The PCR reactions consist of 12.5 µl hot start PCR master mix (Thermo Scientific), 8.5 µl of molecular grade water, 1 µl each forward and reverse primer and 2 µl of genomic DNA (Hashmi et al., 2018). The products were checked on 1% agarose gel and visualized under UV using a gel documentation system (DNr, Bio-Imaging, MiniLumi). The amplified products were sequenced at AgriGenome Pvt. Ltd. (Cochin, India). The quality sequences were assembled with BioEdit version 7.0.0 and deposited in NCBI GenBank.

### Alignment and phylogenetic analyses

For phylogenetic analysis, the majority of species sequences were taken from Zahniser & Dietrich (2013) and Zhaniser & Dietrich (2010) and Du, Liang & Dai (2019). A dataset consisting of the newly sequenced taxa and 76 sequences of Deltocephalinae species. The outgroups consist of two species from Aphrodinae and one species from Euacanthellinae (Table 1).

The histone *H3* and *28S* rDNA sequences aligned separately with the MUSCLE application in MEGA 6 (Tamura et al., 2013a; Tamura et al., 2013b). The aligned sequences of the two gene regions were concatenated into one dataset using Sequence Matrix 1.7.8 (Vaidya, Lohman & Meier, 2011) and obtained NEXUS data block for combined data set as follows: #NEXUS begin data; dimensions ntax = 80 nchar = 6974; format datatype = dna; gap = −; missing = ?; matrix; end;

The NEXUS file used in the phylogenetic analysis deposited in public repository TreeBASE (Study ID S26664; https://treebase.org).

**Table 1:** List of taxa and their accession numbers (H3 and 28S rDNA), used for the phylogenetic. analysis.

**Sl. No**.	**Tribe**	**Species**	**Accession number**	
			**28S**	**Histone H3**
1	Acinopterini	*Acinopterus acuminatus*	JX845484	GU123790
2	Acostemmini	*Acostemma stilleri*	GU123696	GU123791
3	Acostemmini	*Ikelibeloha cristata*	JF835026	JN177306
4	Aphrodinae/Aphrodini	*Aphrodes bicincta*	AF304579	GU123794
5	Aphrodinae/Xestocephalini	*Xestocephalus desertorum*	AF304619	GU123892
6	Arrugadini	*Arrugada affnis*	GU123699	GU123795
7	Athysanini	*Caranavia separata*	GU123710	GU123807
8	Athysanini	*Anoterostemma ivanhoff*	JX845487	JX845528
9	Athysanini	*Athysanus argentarius*	GU123701	GU123797
10	Bahitini	*Kinrentius* sp.	JX845523	JX845549
11	Bahitini	*Bahita* sp.	GU123702	GU123798
12	Bonaspeiini	*Cerus goudanus*	GU123712	GU123809
13	Bonaspeiini	*Renosteria waverena*	GU123772	GU123878
14	Bonaspeiini	*Bonaspeia eriocephala*	JX845521	GU123804
15	Chiasmini	*Nephotettix modulatus*	GU123754	GU123859
16	Chiasmini	*Listrophora styx*	JX845500	JX845539
17	Chiasmini	*Gurawa minorcephala*	JX845495	JX856131
18	Cicadulini	*Cicadula quadrinotata*	GU123717	GU123813
19	Cicadulini	*Proceps acicularis*	JX845511	JX845550
20	Cochlorhinini	*Cochlorhinus pluto*	AF304586	GU123814
21	Deltocephalini	*Deltocephalus* sp.	GU123721	GU123819
22	Deltocephalini	*Paramesodes* sp.	GU123764	GU123868
23	Dorycephalini	*Dorycephalus baeri*	JX845491	JX845532
24	Drabescini	*Bhatia satsumensis*	GU123706	GU123803
25	Drabescini	*Drabescus* sp.	GU123724	GU123824
26	Drakensbergenini	*Drakensbergena retrospina*	GU123725	GU123825
27	Euacanthellinae	*Euacanthella palustris*	GU123728	GU123827
28	Eupelicini	*Eupelix cuspidata*	AF304644	GU123828
29	Eupelicini	*Paradorydium paradoxum*	AF304637	GU123877
30	Faltalini	*Tenucephalus* sp.	GU123781	GU123886
31	Faltalini	*Hecullus bracteatus*	GU123737	GU123841
32	Fieberiellini	*Fieberiella florii*	AF304594	GU123834
33	Goniagnathini	*Goniagnathus guttulinervis*	GU123736	GU123838
34	Hecalini	*Glossocratus afzelii*	GU123735	GU123837
35	Hecalini	*Attenuipyga vanduzei*	AF304653	GU123822
36	Hecalini	*Hecalus viridis*	AF304596	GU123840
37	Hypacostemmini	*Hypacostemma viridissima*	GU123739	GU123843
38	Koebeliini	*Koebelia grossa*	AF304599	GU123846
39	Koebeliini	***Pinapona sinae^a^***	**MN822010 (D2)** **MN822011 (D9-D10)**	–
40	Koebeliini	***Shivapona shivai^a^***	**MN822007 (D2)** **MN822009 (D9-D10)**	**MN816385**
41	Koebeliini	***Sohipona sohii^a^***	**MN824248 (D2)** **MN824250 (D9-D10)**	**MN816387**
42	Limotettigini	*Limotettix striola*	GU123745	GU123850
43	Macrostelini	*Balclutha neglecta*	GU123704	GU123800
44	Macrostelini	*Dalbulus gelbus*	AF304587	GU123818
45	Macrostelini	*Evinus peri*	GU123731	GU123832
46	Magnentiini	*Magnentius clavatus*	JX845503	JX845541
47	Mukariini	*Mukaria maculata*	GU123750	GU123855
48	Mukariini	*Agrica arisana*	GU123779	GU123884
49	Occinirvanini	*Occinirvana eborea*	JX845507	JX845545
50	Opsiini	*Neoaliturus carbonarius*	GU123752	GU123857
51	Opsiini	*Pseudophlepsius binotatus*	JX845512	JX845551
52	Opsiini	*Hishimonus phycitis*	GU123738	GU123842
53	Opsiini	*Japananus hyalinus*	JX845499	JX845538
54	Opsiini	***Libengaia*sp*. ^a^***	**(a) MN820445 (D2)** **(b) MN820441 (D9-D10)**	**MN816383**
55	Opsiini	*Nesophrosyne maritima*	JX845506	JX845544
56	Opsiini	*Opsius versicolor*	GU123756	GU123861
57	Opsiini	*Orosius orientalis*	JX845509	JX845547
58	Opsiini	Opsiini gen. sp.	JX845520	JX845560
59	Opsiini	*Yinformibus menglaensis*	MH260368	MH260369
60	Opsiini	***Vittaliana reticulata^a^***	**(a) MN512542 (D2)** **(b) MN512544 (D9-D10)**	**MK359639**
61	Paralimnini	*Laevicephalus monticola*	GU123744	GU123849
62	Paralimnini	*Aflexia rubranura*	GU123698	GU123793
63	Pendarini	*Bandaromimus parvicauda*	GU123705	GU123802
64	Pendarini	*Tropicanus chiapasus*	GU123784	GU123889
65	Penthimiini	*Penthimidia eximia*	JX845510	JX845548
66	Penthimiini	*Penthimiola bella*	GU123766	GU123871
67	Penthimiini	*Jafar javeti*	JX845498	JX845537
68	Phlepsiini	*Excultanus conus*	GU123732	GU123833
69	Phlepsiini	*Phlepsius intricatus*	GU123768	GU123873
70	Scaphoideini	*Anoplotettix fuscovenosus*	JX845486	JX845527
71	Scaphoideini	*Scaphoideus omani*	JX845513	JX845553
72	Scaphoideini	*Phlogotettix cyclops*	GU123769	GU123874
73	Scaphytopiini	*Scaphytopius frontalis*	JX845514	JX845555
74	Selenocephalini	*Selenocephalus deserticola*	GU123777	GU123881
75	Selenocephalini	*Adama elongata*	GU123694	GU123788
76	Stegelytrini	*Pachymetopius decoratus*	GU123760	GU123864
77	Stenometopiini	*Kinonia elongata*	GU123741	GU123845
78	Stenometopiini	*Stirellus catalinus*	AF304614	GU123882
79	Tetartostylini	*Tetartostylus parabolatus*	GU123782	GU123887
80	Vartini	*Stymphalus rubrolineatus*	GU123778	GU123883

**Notes.**

a Obtained sequences from current study.

Maximum likelihood (ML) analysis of combined gene region (*H3*, *28S* rDNA) were constructed in IQtree v1.4.1 (Nguyen et al., 2015) using the best-fit substitution model automatically selected by the software according to the Bayesian information criterion scores and weights (BIC) with partitions. An ultrafast bootstrap (UFB) (Minh, Nguyen & von Haeseler, 2013) with 1,000 replicates and the SH-like approximate likelihood ratio test (SH-aLRT) (Guindon et al., 2010) and Bayesian-like transformation of aLRT (aBayes) (Anisimova et al., 2011) were used in the analysis to assess branch support and obtained tree was visualized in FigTree v1.4.2.

## Results

**Key to the genera of subtribe Opsiina from India** (Keys modified from El-Sonbati, Wilson & Al Dhafer, 2017).

**Table:** 

1. Subgenital plates and valve fused to form a plate; head, thorax and forewing with bright red, or orange markings....................................................................................*Lampridius* Distant
- Subgenital plates and valve not fused but free; coloration not as above…………..........2
2. Forewings ivory or silvery white, yellow, and brown marks on body, wings at rest with large brown semicircular spot against midlength of commissural margin of forming and conspicuous circular spot along with that of opposite side...................................................................3
- Not like above character ……............................................................................................ 5
3. Aedeagus with 2 or 3 pairs of ventral processes…....................... …*Hishimonoides* Ishihara
- Aedeagus with a pair of ventral processes ………………………………........................... .4
4. Aedeagus with atrium not extending ventrad of shafts…………...*Hishimonus* Ishihara
- Aedeagus with atrium extending ventrad of shafts ………................................*Litura* Knight
5. Aedeagus with unpaired ventral process bifurcate in apical half…..*Libengaia* Linnavuori
- Aedeagus without unpaired ventral process ………………………........ ……………6
6. Aedeagus with basal processes ....................................................................................................7
- Aedeagus without basal processes..................................................................................................8
7. Anterior margin of head rounded or slightly produced, not concave; aedeagus with one or two pairs of processes, arising from socle or from mid-length of shaft.............*Opsius* Fieber
- Anterior margin of head slightly produced, slightly concave; aedeagus with one pair of processes, arising only from mid-length of shaft ……....................................... *Vittaliana***gen. nov.**
8. Fore wing, vertex, pronotum and scutellum with dark brown vermiculate line……........…9
- Not like above characters ………..........................................................................................10
9. Vertex narrow basally, diamond shaped; compound eyes very close to each other posteriorly (fig40; Viraktamath & Anantha Murthy, 1999); aedeagal shafts with apical an elongated recurved process........................................................................................................*Pugla* Distant
- Vertex not narrow basally, not diamond shaped; compound eye are not close to each other posteriorly; aedeagal shafts without apical process …………………………*Orosius* Distant
10. Crown, pronotum and scutellum with irregular red markings; pronotum without lateral carina; aedeagus with shafts fused at basal 0.33x (fig36; Viraktamath & Anantha Murthy, 1999) ……...................................................................................................................*Masiripius* Dlabola
- Crown, pronotum and scutellum without irregular red markings; pronotum with lateral carina; aedeagus with shafts seperated from base (fig26; Viraktamath & Anantha Murthy, 1999) ...............................................................................................................................*Japananus* Ball

### Taxonomy

**Table:** 

**Figure 1:**
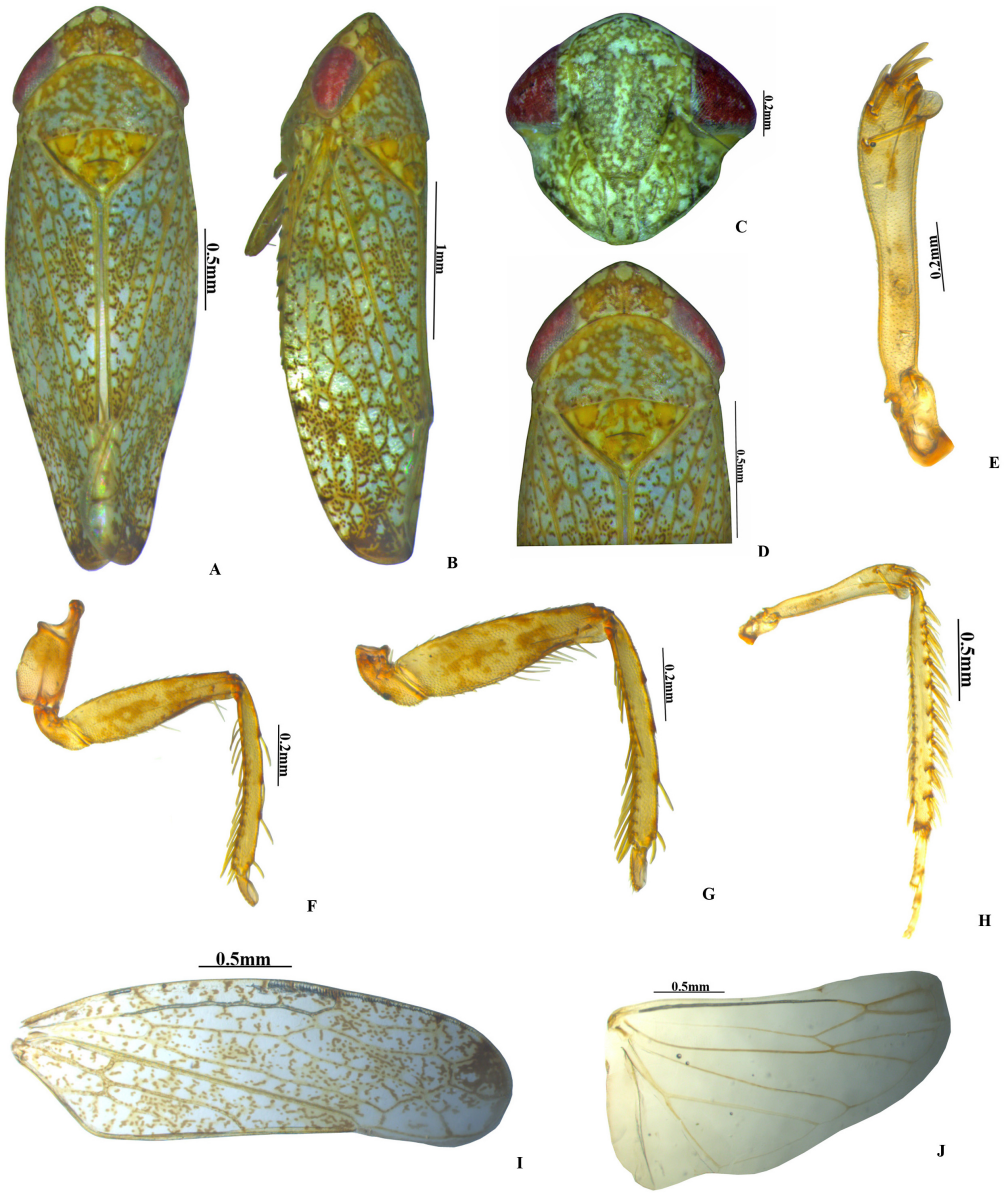
* Vittaliana reticulata* Sunil and Meshram gen. nov., sp. nov. Male. (A) Habitus dorsal; (B) Habitus lateral; (C) Face; (D) Pronotum; (E) Metathoracic femur; (F) Foreleg; (G) Midleg; (H) Hindleg; (I) Forewing; (J) Hindwing.

**Figure 2:**
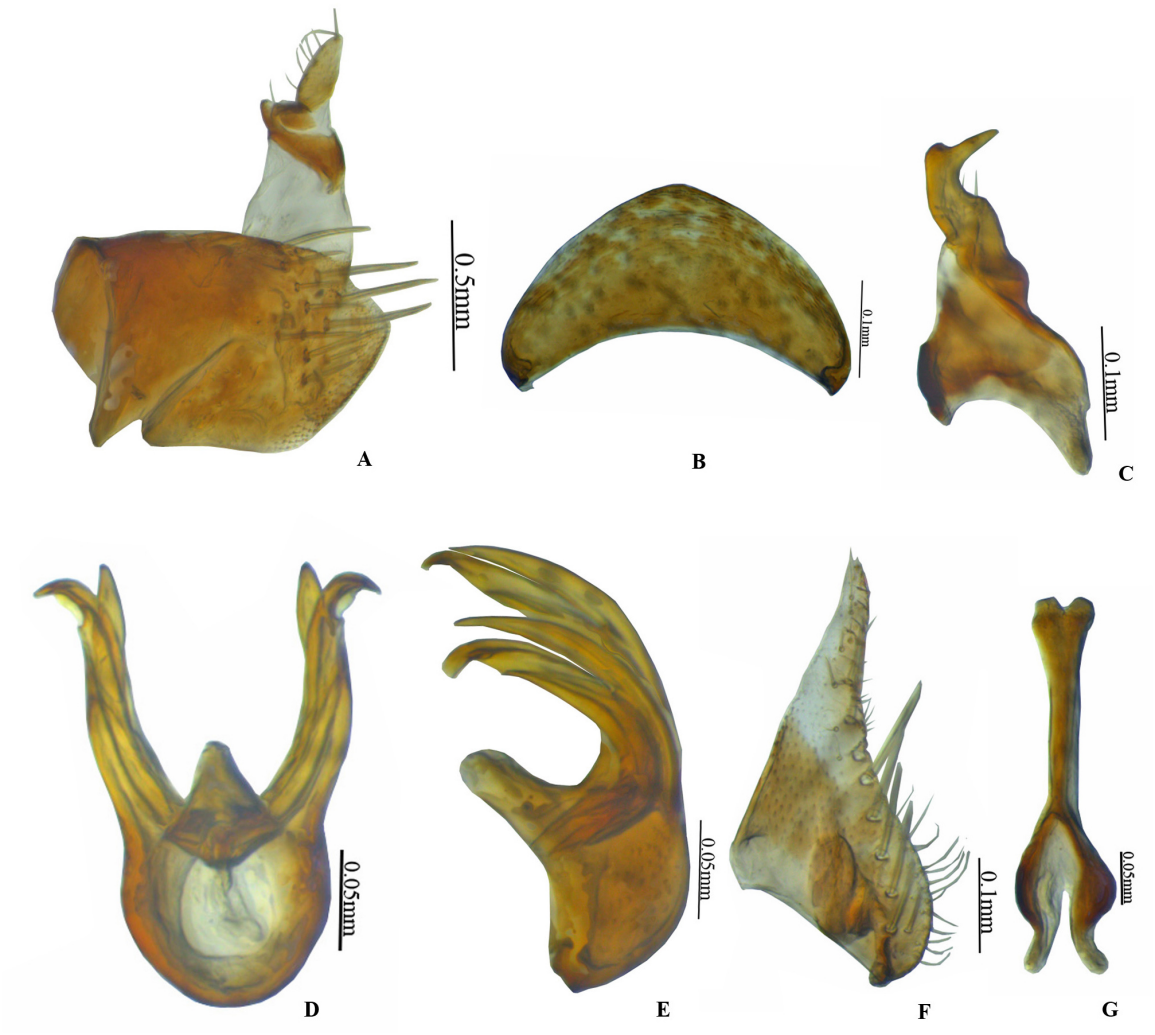
* Vittaliana reticulata* Sunil and Meshram gen. nov., sp. nov. Male genitalia. (A) Pygofer lateral; (B) Valve; (C) Style; (D) Aedeagus ventral; (E) Aedeagus lateral; (F) Subgenital plate; (G) Connective.

**Figure 3:**
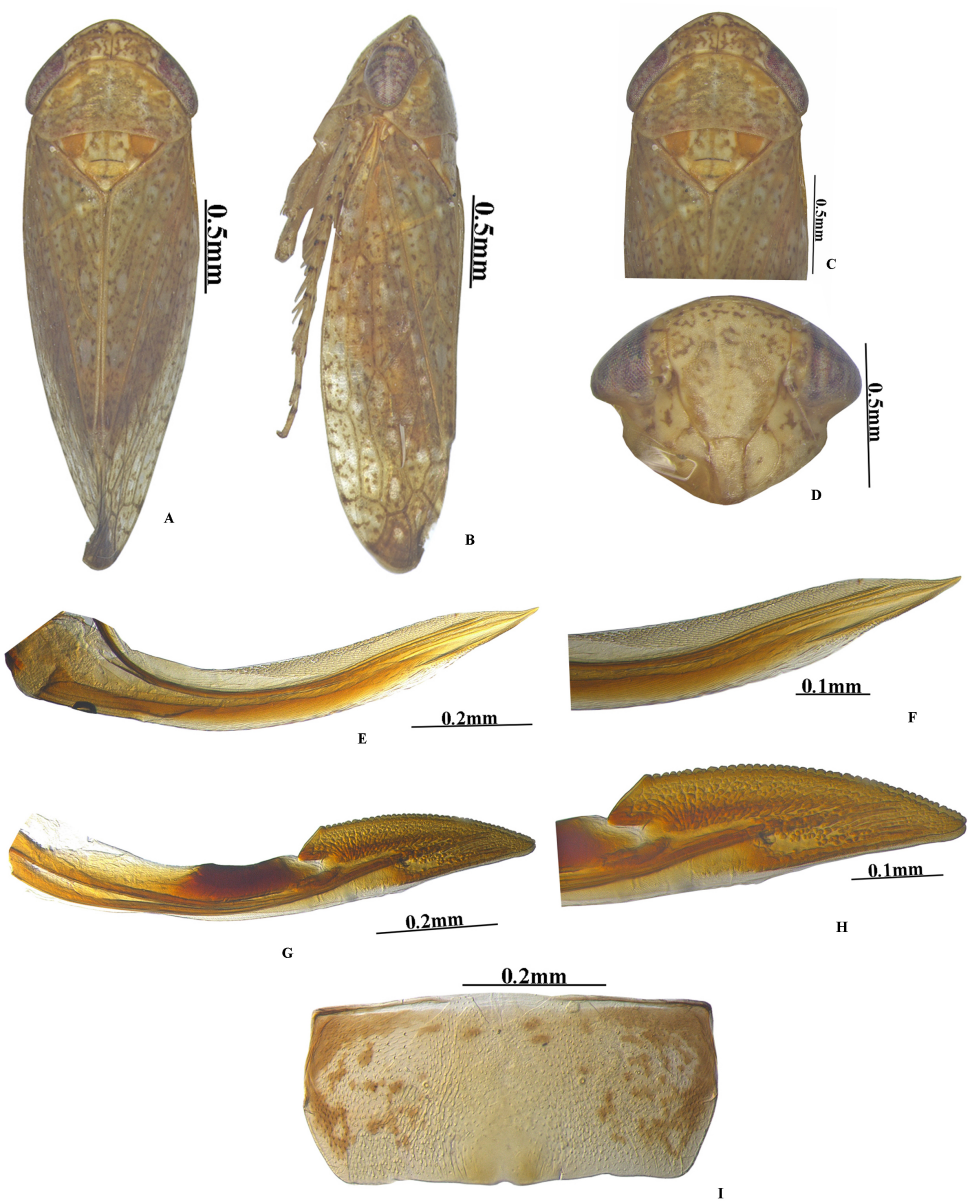
*Vittaliana reticulata* Sunil and Meshram gen. nov., sp. nov. Female. (A) Habitus dorsal; (B) Habitus lateral; (C) Pronotum; (D) Face; (E) Ist Valvulae; (F) Ist Valvulae apical view; (G) IInd Valvulae; (H) IInd valvulae apical view; (I) VIIth sternite.

**Family Cicadellidae**
**Subfamily Deltocephaliae**
**Tribe Opsiini**
***Vittaliana*gen. nov. Sunil and Meshram (Figs. 1–3)**
urn:lsid:zoobank.org:act:51DA3683-0359-444F-8C11-F630518D8506

**Type species**: ***Vittaliana reticulata*** **sp. nov. Sunil and Meshram [Figs. 1A–1J, 2A–2G, 3A–3H]**

### Diagnosis

This genus is placed in the subtribe Opsiina of Opsiini based on all these characters macropterous; ovipositor not protruding far beyond pygofer apex and subgenital plates with a lateral row of macrosetae; aedeagal shafts divided near to base. The new genus can be differentiated from all related genera in this subtribe by a combination of the following characters: Body and face whitish with yellow, and brown mottling; anterior margin of crown with five white patches, slightly produced, slightly concave; pronotum with concave posterior margin; aedeagal shafts arising from near to base, aedeagus distinctive outward curved apically, without processes arising from base, with a pair of medial processes arising from mid-length of shafts, apex without processes.

### Description

**Colour.** Body and face whitish with yellow and brown mottling. Crown anterior margin with five white patches (Fig. 1D). Pronotum brown mottled with white patches. Scutellum yellow with an orange basal triangle (Fig. 1D). Eyes red; forewing white speckled with brown patches (Fig. 1B).

**Body length.** Male 3.6 mm long; 1.4 mm wide across eyes. Female 3.8 mm long; 1.46 mm wide across eyes.

**Head.** Anterior margin of head slightly produced, head in dorsal view as wide as pronotum; crown length 2/3rd as long as median length of the pronotum, anterior margin produced with concave posterior margin; face with brown and white irregular mottling; ocelli small, close to eyes on anterior margin of crown; clypellus 2.8x as long as wide; gena obtusely incised laterally (Fig. 1C).

**Thorax.** Pronotum anterior margin convex, 2x as broad as long, hind margin slightly concave; scutellum 1.5x as broad as long, and 0.6x as long as width of pronotum, with distinct scutoscutellar suture (Fig. 1D). Forewing elongate, veins raised, three subapical and four apical cell, claval vein raised with 2 crossveins, appendix extended around the apex (Fig. 1I). Hind wing veination complete, appendix broad (Fig. 1J).

**Legs.** Prothoracic femur with AM1 seta only; intercalary row with one row of more than 5 fine setae; AV row with 2–3 macrosetae, AV1 seta long; AD setae small and sparsely arranged. Prothoracic tibia on dorsal surface rounded, AD row with 4–5 setae long, distributed widely; AV setae moderately dense and long (Fig. 1F). Mesothoracic femur with AD setae small; AV row with basal half short setae and rest with macrosetae; AM seta present; intercalary row with one row of more than 5 fine setae (Fig. 1G). Metathoracic femur with setal formula 2+2+1; lateral surface area broadened distally; metathoracic tibia flattened, tibial row AD setae long and densely arranged, PD with long macrosetae placed equidistantly, AV setae small and densely arranged, PV with macrosetae moderately arranged, Metatarsomere I length equals to tarsomere II and tarsomere III combined (Fig. 1H).

**Male genitalia.** Pygofer longer than wide with macrosetae in midlateral region, with anal tube long, 3/4th membranous from the base (Fig. 2A). Valve triangular with broad base (Fig. 2B). Subgenital plate triangular, basally broad, posterior half gradually narrowed towards apex, with 7–8 submarginal macrosetae, 7 microsetae medially on distal 2/3rd (Fig. 2F). Style broadly bilobed basally, subapical angle not prominent with few setae (Fig. 2C). Connective Y-shaped, stem 1.3x as long as arms (Fig. 2G). Aedeagus with well-developed dorsal apodeme, with a pair of medial processes arising from mid-length of shafts, gonopores subapical (Fig. 2D).

**Female genitalia.** Female seventh sternite trapezoid in shape, sternite 2x as wide as median length, hind margin with sinuate with shallow notch medially (Fig. 3I); first pair of valvulae wider beyond the base and narrowed at apex, with irregular sculpture on apical 1/2th, dorsal hyaline area restricted to basal half (Fig. 3F); second pair of valvulae, with small teeth and sculpting on apical half (Fig. 3H).

### Etymology

This genus was named after the place of collection, Vittal in Kerala, India.

### Distribution

Kerala, Karnataka, India

**Table:** 

***Vittaliana reticulata*sp. nov. Sunil and Meshram**
urn:lsid:zoobank.org:act:E690A7AF-7FCD-4460-93BC-1274D932C5F4

**Diagnosis.** In addition to generic character, the specific characters for this species are: anterior margin of crown slightly produced with five white patches (Fig. 1D), face with brown and white irregular marking (Fig. 1C). Pygofer with a group of macrosetae confined to the mid-lateral region (Fig. 2A). Style with beak-like apophysis, directed posteriorly (Fig. 2C). Connective Y- shaped, stem longer than arms (Fig. 2G). Aedeagal gonopore opens subapically on ventral margin with constriction (Fig. 2D). Seventh sternite 2x as wide as median length, hind margin with shallow notch medially (Fig. 3I).

### Description.

**Colour.** Body and face whitish with yellow and brown mottling; anterior margin of crown with five white patches (Fig. 1D). Pronotum brown mottled with white patches. Scutellum yellow with an orange basal triangle. (Fig. 1D); fore wing white speckled with brown patches (Fig. 1B).

Anterior margin of head slightly produced, head in dorsal view as wide as pronotum (Fig. 1D). Ocelli small, close to eyes on anterior margin of crown; clypellus 2.8x as long as wide. Gena obtusely incised laterally (Fig. 1C). Pronotum anterior margin convex, 2x as broad as long; scutellum 1.5x as broad as long with distinct scutoscutellar suture (Fig. 1D). Fore wing macropterous, veins raised, appendix expanded around the apex (Fig. 1I).

**Male genitalia.** Pygofer longer than wide with a group of macrosetae confined to mid-lateral region (Fig. 2A). Valve 2x as wide at base as long medially (Fig. 2B). Subgenital plate triangular, broad at base slightly tapering towards the apex with 7–8 submarginal macrosetae (Fig. 2F). Style bilobed basally, subapical angle not prominent with beak-like apophysis, directed posteriorly (Fig. 2C). Connective Y- shaped, stem longer than arms (Fig. 2G). Aedeagus with a pair of medial processes arising from mid-length of shafts, gonopore opens subapically on ventral margin with constriction (Fig. 2D).

**Female genitalia.** Seventh sternite 2x as wide as median length, hind margin with shallow notch medially (Fig. 3I). First pair of valvulae with an irregular sculpture on apical 1/2th (Fig. 3F). Second pair of valvulae with small teeth and sculpting on apical half (Fig. 3F).

### Type material

#### Holotype

INDIA •♂; Kerala: Kasargod: Vittal CPCRI, 12°46′11.87″N, 75°06′47.91″E; 80m MSL; 24.I.2016; Anooj and Twinkle; mercury vapour lamp; RRS2 (NPC).

#### Paratypes

INDIA•1♂, 1♀; Kerala: Nilambur, 11.2794° N, 76.2398° E; 20.XI.2008; 200m MSL; Murthy S; mercury vapour lamp; RRS3 (♂), RRS4 (♀) (NPC); INDIA•1 ♀; Karnataka: Mudigere, 13.1365°N, 75.6403°E; 970m; 25.V.1976; mercury vapour lamp; RRS5 (NPC).

#### Etymology

The species name, “*reticulata*” is based on the reticulated forewing venation.

#### Molecular analysis

**Figure 4:**
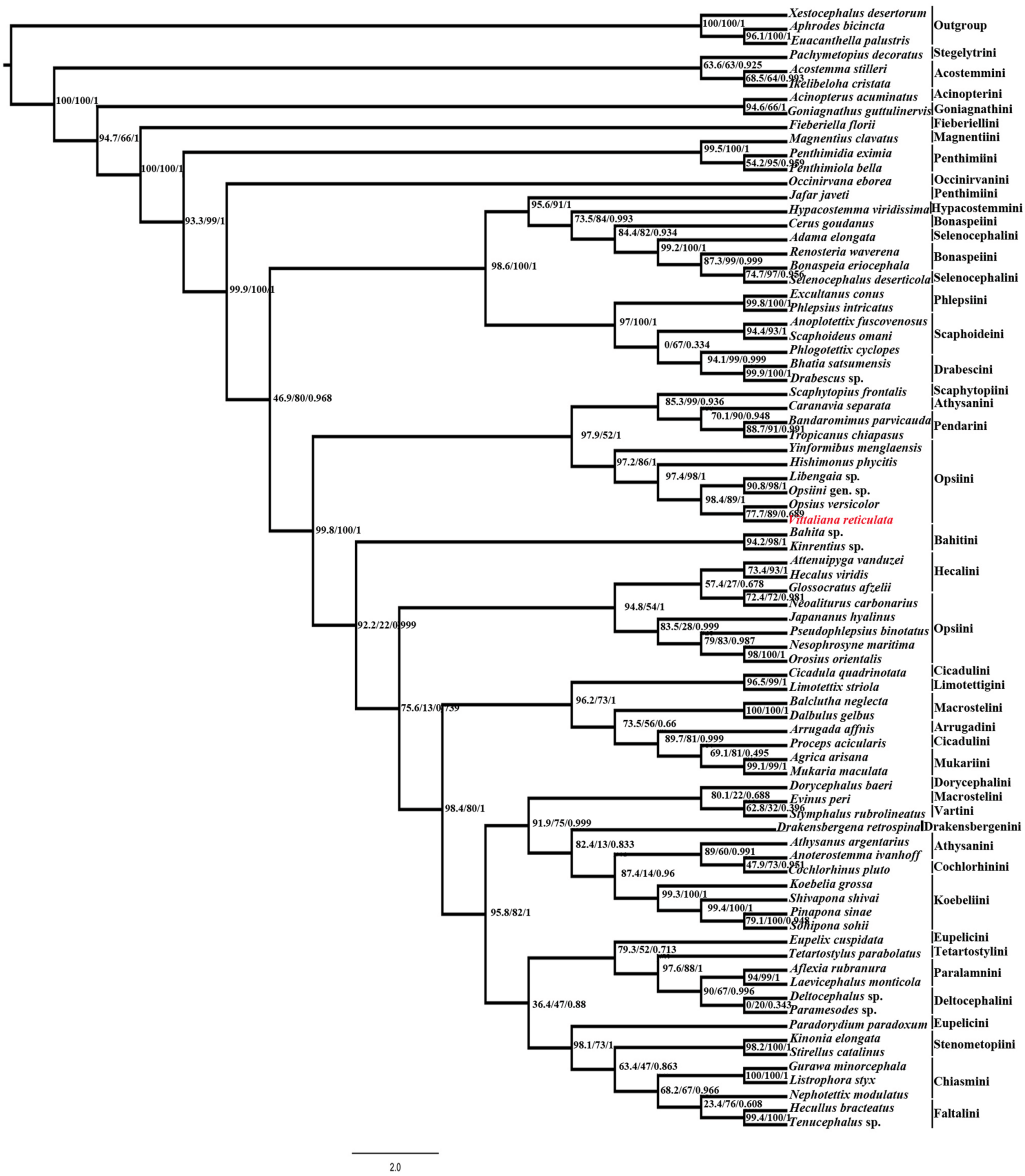
Maximum-likelihood (ML) tree estimated from the combined dataset (Histone *H3*, *28S* rDNA). At each node, values indicate ML support and Bayesian-like transformation of aLRT (aBayes): SH-like appropriate likelihood ratio test (SH-aLRT)/Ultrafast bootstrap (UFB)/ Bayesian-like transformation of aLRT (aBayes) values.

Maximum likelihood (ML) analysis using IQtree v1.4.1 of the 80 taxa and 6074bp aligned nucleotide position of combined Histone *H3* and *28S* rDNA (D2 & D9-D10 region) yielded maximum likelihood phylogenetic tree (Fig. 4) with good SH-like approximate likelihood ratio test (SH-aLRT), ultrafast bootstrap (UFB) and Bayesian-like transformation of aLRT (aBayes). Our new species *Vittaliana reticulata* is sister to clade *Opsius versicolor* (Distant), *Opsiini* gen. sp., *Libengaia* sp., *Hishimonus phycitis* (Distant) and *Yinfomibus menglaensis* Du, Liang & Dai. Among them *V. reticulata* closely associated with *Opsius versicolor* with good SH-aLRT score (77.7), UFB (89) and moderate aBayes (0.689), indicates that the new genus belongs to the tribe Opsiini under subfamily Deltocephalinae. All included 11 species of Opsiini including new genus, form two clades in the phylogenetic tree resolve as paraphyletic with respect to clade Pendarini + Athysanini + Scaphytopiini in one clade and with Hecalini in another clade with SH-aLRT (>90), low UFB (>50) and aBayes (1).

## Discussion

The most important diagnostic character to the tribe Opsiini is the presence of bifurcate aedeagus with paired shafts and gonopores, although this character is also found in *Alocoelidia* of the tribe Acostemini (Zahniser & Nielson, 2012) and some genera of the tribe Scaphytopiini and Mukariini (Zahniser & Dietrich, 2013). *Vittaliana* gen. nov. the best fits into the tribe Opsiini because it lacks the diagnostic morphological characters that define the above tribes, i.e., Mukariini have a depressed body form, with the face nearly the horizontal and the anterior margin usually transversely carinate, and Scaphytopiini has the head strongly produced with gena extended onto dorsum behind eyes (Du, Liang & Dai, 2019). *Vittaliana* gen. nov. has the face oblique, not strongly depressed in profile, the stem of the connective is long, as in many other Opsiini and its aedeagal shafts arising from near to base, aedeagus distinctive outward curved apically, without processes arising from the base with a pair of medial processes arising from mid-length of shafts, apex without processes, and gonopore open subapically. Maximum likelihood (ML) analysis using combined data of Histone *H3* and *28S* rDNA (D2 & D9-D10 region) yielded a maximum likelihood phylogenetic tree. The new genus *Vittaliana* is sister to clade *Opsius versicolor*, *Opsiini* gen. sp., *Libengaia* sp., *Hishimonus phycitis,* and *Yinfomibus menglaensis* with good support branch, this resolve the placement of new genus in the tribe Opsiini. In the phylogenetic tree, 11 species of Opsiini including new genus form two clades, and connecting with species of Pendarini, Athysanini, Scaphytopiini and Hecalini with SH-aLRT (>90), low UFB (>50) and aBayes (1), this confirms the species of Opsiini resolve as paraphyletic. Our study is not consistent with the previous phylogenetic study of new genus *Yinformibus* Du, Liang & Dai (2019) based on combined Histone *H3* and *28S* rDNA resolve tribe Opsiini as monophyletic with moderate bootstrap support (85%). In contrast, our study combined dataset of histone *H3* and *28S* rDNA resolve the tribe opsiini as paraphyletic with low UFB (>50) and aBayes (1) which may be due to the addition of more members of Opsiini in the phylogenetic analysis may diverge as paraphyletic. However, our study consistent with the previous phylogenetic analysis of Deltocephalinae including combined data from the *28S* rDNA, Histone *H3* and morphological data (Zahniser & Dietrich, 2013) with approximately similar (<50% ML) bootstrap but the more detailed phylogenetic analysis is needed. Inclusion of more species of Opsiini and more gene addition may resolve the relationship of Opsiini in highly diverse Deltocephalinae.

## Conclusion

The present study reveals that new genus *Vittaliana reticulata* gen. nov., sp. nov. belongs in the tribe Opsiini and subtribe Opsiina by morphological characters and molecular phylogenetic analysis. This genus differs from closely related genera by morphological characters and based on available molecular data analysis establish that this genus and species was closely related to the type genus of Opsiini and also indicated that the tribe Opsiini is paraphyletic. However further study is needed by adding more genes to see its evolutionary significance.

##  Supplemental Information

10.7717/peerj.9515/supp-1 Supplemental Information 1 Maximum-likelihood (ML) tree estimated from the combined dataset (Histone *H3*, *28S* rDNA) At each node, values indicate ML support and Bayesian posterior probability (BPP). SH-like appropriate likelihood ratio test (SH-aLRT)/ Bayesian posterior probability (BPP)/Ultrafast bootstrap (UFB) values.

## Additional Information and Declarations

### Data Availability

The following information was supplied regarding data availability: *Vittaliana reticulata** sequences are available at GenBank: MN512542 (28S Gene D2 region), MN512544 (28S Gene D9-D10 region) and MK359639 (Histone). Type materials are deposited in National Pusa Collection, IARI, New Delhi, with repository number RRS2, RRS3, RRS4 and RRS5. Data is also available at TreeBASE (Study ID S26664; https://treebase.org).

### New Species Registration

The following information was supplied regarding the registration of a newly described species: Publication LSID: urn:lsid:zoobank.org:pub:228D17FC-590C-41C7-9434-60554F753DBA. Vittaliana LSID: urn:lsid:zoobank.org:act:51DA3683-0359-444F-8C11-F630518D8506 Vittaliana reticulata LSID: urn: lsid:zoobank.org:act:E690A7AF-7FCD-4460-93BC-1274D932C5F4.
