# Supplementary figures and images for: A new genus Vittaliana belonging to the tribe Opsiini (Hemiptera: Cicadellidae) from India and its molecular phylogeny
Source: PeerJ. 2020 Aug 27;8:e9515. doi: 10.7717/peerj.9515 (PMC7456527; doi:10.7717/peerj.9515)

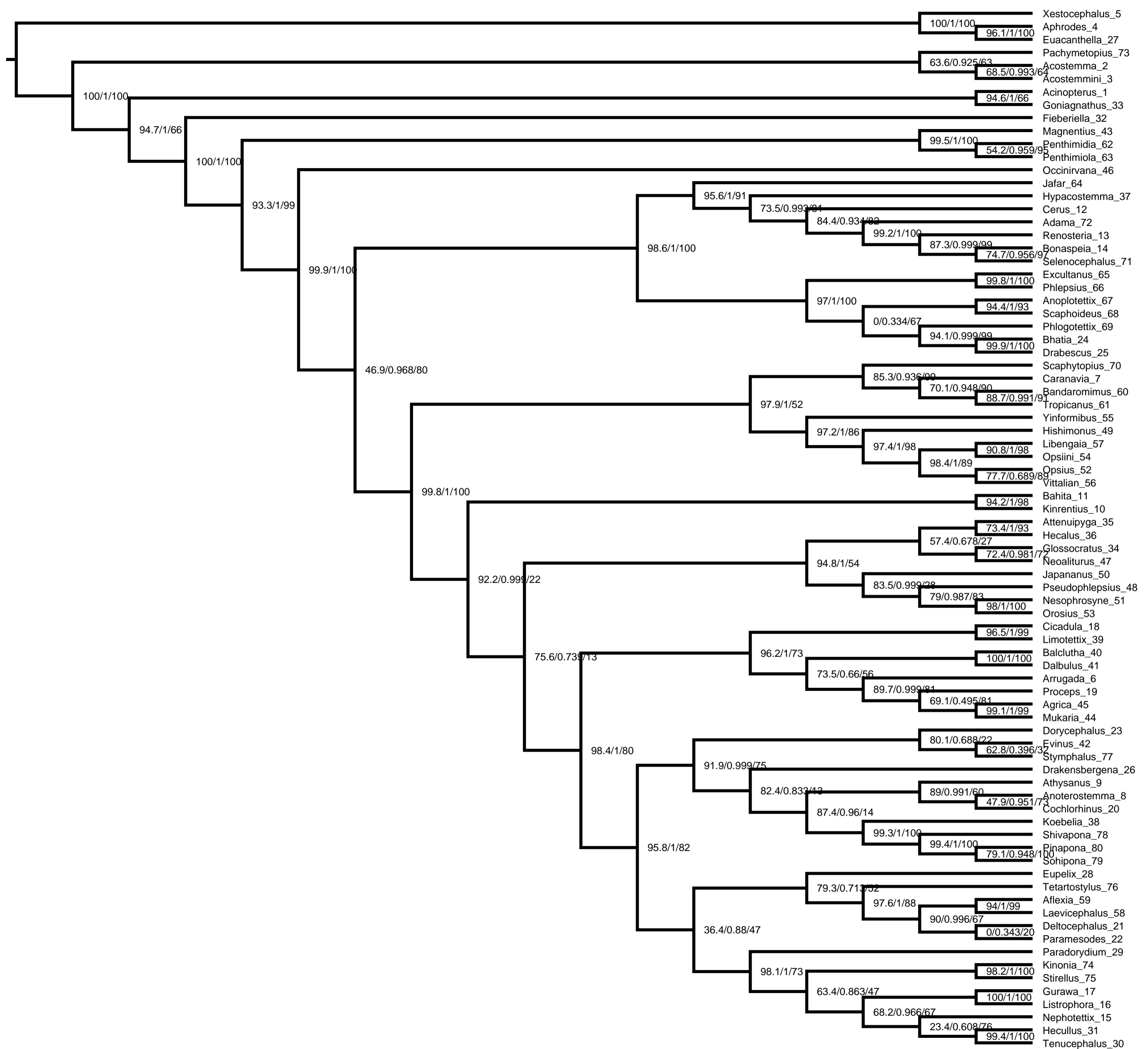

Supplement: Supplemental Information 1 — At each node, values indicate ML support and Bayesian posterior probability (BPP). SH-like appropriate likelihood ratio test (SH-aLRT)/ Bayesian posterior probability (BPP)/Ultrafast bootstrap (UFB) values. [file peerj-08-9515-s001.pdf]
